# Supplementary material for: The causal role of circulating inflammatory markers in osteoporosis: a bidirectional Mendelian randomized study
Source: Front Immunol. 2024 Jul 18;15:1412298. doi: 10.3389/fimmu.2024.1412298 (PMC11291241; doi:10.3389/fimmu.2024.1412298)
Supplement: Supplementary file 1 [file DataSheet_1.zip › supplementary material/OSTEOPOROSIS-CIM/GCST90274829.tsv.gz_leave_one_out.pdf]

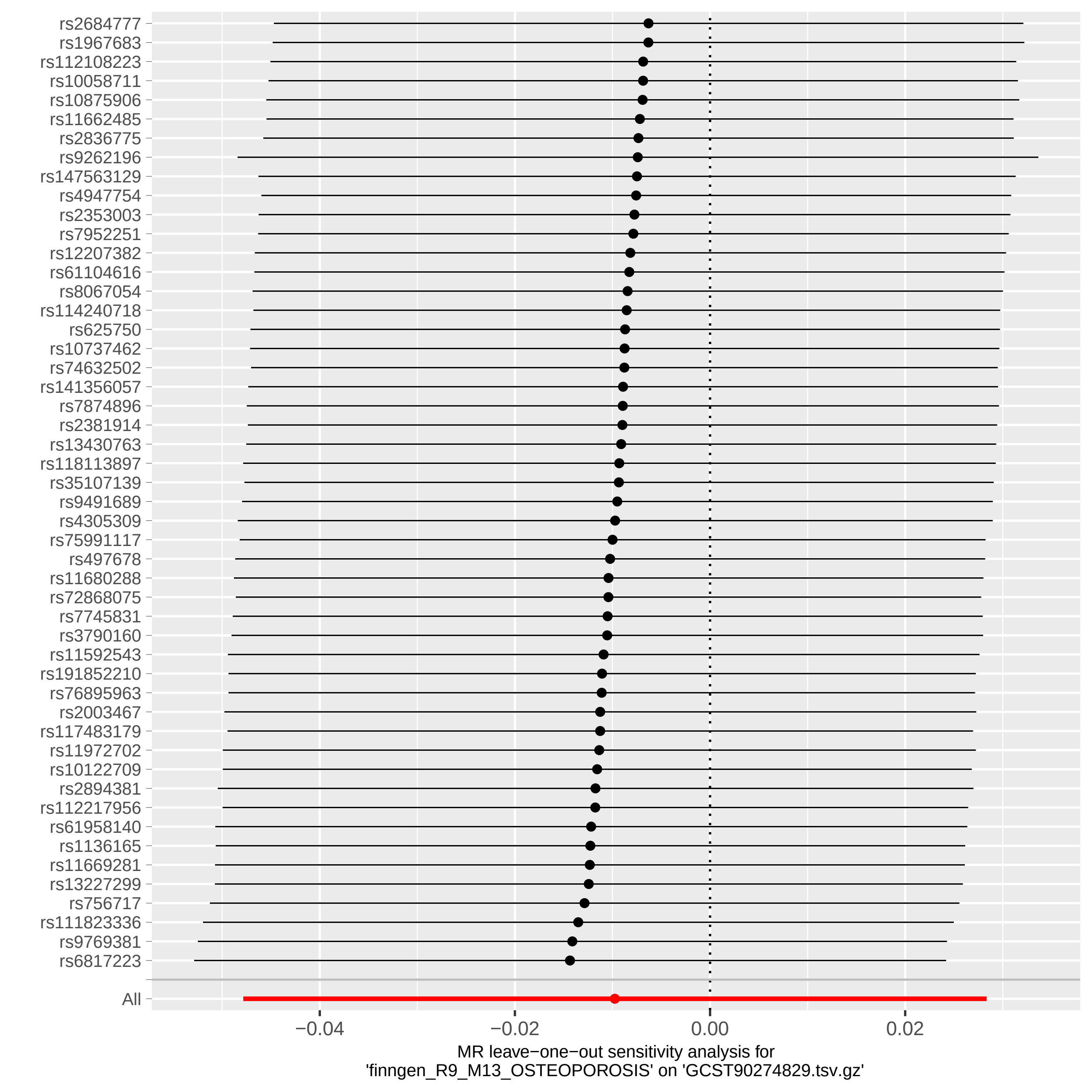
rs2684777
rs1967683
rs112108223
rs10058711
rs10875906
rs11662485
rs2836775
rs9262196
rs147563129
rs4947754
rs2353003
rs7952251
rs12207382
rs61104616
rs8067054
rs114240718
rs625750
rs10737462
rs74632502
rs141356057
rs7874896
rs2381914
rs13430763
rs118113897
rs35107139
rs9491689
rs4305309
rs75991117
rs497678
rs11680288
rs72868075
rs7745831
rs3790160
rs11592543
rs191852210
rs76895963
rs2003467
rs117483179
rs11972702
rs10122709
rs2894381
rs112217956
rs61958140
rs1136165
rs11669281
rs13227299
rs756717
rs111823336
rs9769381
rs6817223
All
−0.04
−0.02
0.00
0.02
MR leave−one−out sensitivity analysis for
'finngen_R9_M13_OSTEOPOROSIS' on 'GCST90274829.tsv.gz'
